# Editorial: Advances in Computational Neuroscience

**DOI:** 10.3389/fncom.2021.824899

**Published:** 2022-01-24

**Authors:** Thomas Nowotny, Sacha J. van Albada, Jean-Marc Fellous, Julie S. Haas, Renaud B. Jolivet, Christoph Metzner, Tatyana Sharpee

**Affiliations:** ^1^School of Engineering and Informatics, University of Sussex, Brighton, United Kingdom; ^2^Jülich Research Centre, Institute of Neuroscience and Medicine (INM-6) and Institute for Advanced Simulation (IAS-6) and JARA-Institute Brain Structure-Function Relationships (INM-10), Jülich, Germany; ^3^Department of Biology, Faculty of Mathematics and Natural Sciences, Institute of Zoology, University of Cologne, Cologne, Germany; ^4^Departments of Psychology and Biomedical Engineering, University of Arizona, Tucson, AZ, United States; ^5^Department of Biological Sciences, Lehigh University, Bethlehem, PA, United States; ^6^Maastricht Centre for Systems Biology, Maastricht University, Maastricht, Netherlands; ^7^Department of Electrical Engineering and Computer Science, Institute of Software Engineering and Theoretical Computer Science, Technische Universität Berlin, Berlin, Germany; ^8^Department of Child and Adolescent Psychiatry, Charité Universitätsmedizin, Berlin, Germany; ^9^School of Physics, Engineering and Computer Science, University of Hertfordshire, Hatfield, United Kingdom; ^10^Computational Neurobiology Laboratory, Salk Institute for Biological Studies, La Jolla, CA, United States; ^11^Department of Physics, University of California, San Diego, La Jolla, CA, United States

**Keywords:** computational neuroscience, computational modeling, data analysis, brain circuits, neuronal networks, neural dynamics

The 28th Annual Computational Neuroscience Meeting CNS^*^2019 took place from 13 to 17 July 2019 in the city of Barcelona. The conference encompassed a wide diversity of Research Topics and welcomed participants from around the world, with keynotes on “Brain networks, adolescence, and schizophrenia” by Professor Ed Bullmore, “Neural circuits for mental simulation” by Professor Kenji Doya, “One network, many states: varying the excitability of the cerebral cortex” by Professor Maria Sanchez-Vives, and “Neural circuits for flexible memory and navigation” by Professor Ila Fiete. The present Research Topic, “*Advances in Computational Neuroscience*,” contains some of the leading-edge Computational Neuroscience research presented and discussed at the conference.

Like CNS^*^2019, the articles in this Research Topic reflect the diversity and richness of computational neuroscience research, expanding from subcellular scales to networks, from biological details to *in silico* technology, and from computational methods to brain theory.

At the sub-neuronal level, in “ROOTS: An algorithm to generate biologically realistic cortical axons and an application to electroceutical modeling,” Bingham et al. develop computational methods for building more accurate computational models, extending the capability of generative methods for producing neuronal morphology of highly branched cortical axon terminal arbors. In a similar domain, in “Serotonergic Axons as Fractional Brownian Motion Paths: Insights into the Self-organization of Regional Densities,” Janušonis et al. describe how a computational model based on reflected Fractional Brownian Motion can generate steady-state distributions that approximate the experimentally observed serotonergic fiber distributions in physical brain sections. Gontier and Pfister, in “Identifiability of a binomial synapse,” expand model principles by introducing a definition of when a statistical model is practically identifiable and apply this concept to models of synapses. Felton et al. in “Assessing the Impact of I_h_ Conductance on Cross-Frequency Coupling in Model Pyramidal Neurons,” analyze the role of the hyperpolarization-activated mixed cation current (I_h_) in the dynamical phenomenon of cross-frequency coupling. In a similar vein, Mergenthal et al. in “A Computational Model of the Cholinergic Modulation of CA1 Pyramidal Cell Activity,” present a computational model of pyramidal cells that includes unprecedented detail on receptor activations in response to changes in extracellular acetylcholine concentration, and describe their effects on cellular excitability and downstream intracellular calcium dynamics.

Other authors work at the network level. In “From topological analyses to functional modeling: the case of hippocampus,” Dabaghian presents an approach to network modeling in which a combination of topological analyses provides insights into information processing in mammalian hippocampus. Similarly, Hasanzadeh et al. in “Necessary Conditions for Reliable Propagation of Time-Varying Firing Rate,” investigate which network properties in feed-forward networks allow stable propagation of asynchronous spikes, while Zirkle and Rubchinsky, in “Spike-Timing Dependent Plasticity Effect on the Temporal Patterning of Neural Synchronization,” investigate in a small network model how plasticity of synapses can alter synchronization dynamics and induce intermittent synchronization akin to experimental observations. Tian and Zhou, on the other hand, focus more on simulation algorithms in “Exponential Time Differencing Algorithm For Pulse-coupled Hodgkin-Huxley Neural Networks.”

A third group of works relates computational modeling with higher-level experimental observations. Endo et al. in “Evaluation of resting spatio-temporal dynamics of a neural mass model using resting fMRI connectivity and EEG microstates,” use the Larter-Breakspear neural mass model to relate to both, fast EEG/MEG microstates and slow fluctuations observed with fMRI. Relatedly, Don et al. in “Topological View of Flows inside the BOLD Spontaneous Activity of the Human Brain,” use computational topology of data and discover previously unknown vortex structures in activated brain regions. Finally, within this category, Li et al. in “Effects of cholinergic neuromodulation on thalamocortical rhythms during NREM sleep: a model study,” study a new model of the thalamo-cortical network with cholinergic modulation that exhibits the hallmarks of NREM sleep and allows formulating hypotheses for the role of this important neuromodulator in generating these hallmarks.

Other works are inspired by principles from statistical physics and information theory. For instance, in “A computational framework for controlling the self-restorative brain based on the free energy and degeneracy principles,” Park and Kang speculate about a method based on the free energy principle to identify ways of using the brain's self-restoration capabilities to induce desired changes in brain activity. Sorooshyari et al. in “Object Recognition at Higher Regions of the Ventral Visual Stream via Dynamic Inference,” hypothesize about the computations performed by the ventral visual stream during object recognition based on dynamic inference, and present simulations of object identification by inferior temporal cortex. On the other hand, Yamakou et al. in “Optimal self-induced stochastic resonance in multiplex neural networks: electrical vs. chemical synapses,” are inspired by stochastic resonance, and investigate in a computational model the differential roles of electrical and chemical synapses and their properties for supporting this mechanism. Finally, Ludl and Soriano, in “Impact of physical obstacles on the structural and effective connectivity of *in silico* neuronal circuits,” take us all the way to cultured brain circuits and find that in their simulations physical obstacles placed into the path of neurons growing on silicon substrates lead to the formation of local effective microcircuits.

While the breadth and diversity of the submitted work could hardly be larger, there are also striking commonalities. Many of the works draw on a rich set of advances from several disciplines. This may reflect the typically multi-disciplinary background of the author teams, but we believe is also a sign of the future of the maturing field of computational neuroscience, which has long since moved beyond building models of isolated aspects of brains, computation, or behavior. Our future lies in the cross-disciplinary amalgamation of methods, theories, models, and experimental data. In this spirit, we will continue to be an open and welcoming community as the Computational Neuroscience Meeting enters its 4th decade.

## Author Contributions

TN wrote the initial draft. SA, J-MF, JH, RJ, CM, and TS edited and approved the manuscript.

## Conflict of Interest

The authors declare that the research was conducted in the absence of any commercial or financial relationships that could be construed as a potential conflict of interest.

## Publisher's Note

All claims expressed in this article are solely those of the authors and do not necessarily represent those of their affiliated organizations, or those of the publisher, the editors and the reviewers. Any product that may be evaluated in this article, or claim that may be made by its manufacturer, is not guaranteed or endorsed by the publisher.

